# Interactions between upper limb asymmetry, isokinetic shoulder function, and sprint performance in wheelchair rugby: a preliminary study

**DOI:** 10.3389/fspor.2026.1890052

**Published:** 2026-10-22

**Authors:** Karina Santos Guedes de Sá, Hugo Vinícius de Oliveira Silva, Edison Ramos Cavalari, Cláudia Regina Cavaglieri, José Irineu Gorla

**Affiliations:** 1 School of Physical Education, State University of Campinas (UNICAMP), Campinas, Brazil; 2 Multidisciplinary Center for Paralympic and Parasports Studies, State University of Campinas (UNICAMP), Campinas, Brazil; 3 North Paraná University (UNOPAR), Londrina, Brazil

**Keywords:** muscle imbalance, paralympic athletes, peak torque, shoulder biomechanics, wheelchair propulsion

## Abstract

**Background:**

Wheelchair rugby (WR) athletes often present neuromuscular asymmetries due to the nature of their impairments and sport-specific demands. Understanding whether these asymmetries influence performance is important for characterizing functional profiles and guiding training strategies. Therefore, the aim of this study was to determine whether muscle asymmetry and balance influence the relationship between isokinetic muscle function variables and sprint performance in WR athletes.

**Methods:**

Nine elite WR athletes underwent isokinetic testing of shoulder flexion, extension, abduction, and adduction at 180°/s to determine peak torque normalized by body weight (PT/BW), bilateral asymmetry based on PT/BW, and agonist/antagonist ratios. Sprint performance was assessed using a sprint test. Spearman correlations and univariate regression models evaluated associations between isokinetic variables, bilateral asymmetry (continuous variable representing the magnitude of the percentage difference in peak torque between the limbs), muscle balance (agonist and antagonist ratio), and mean sprint speed.

**Results:**

The magnitude of bilateral peak torque differences varied across athletes and shoulder movements. Shoulder adduction showing significantly greater asymmetry than abduction (*p* = 0.009). All athletes demonstrated marked agonist/antagonist imbalances. Shoulder extension PT/BW was significantly correlated with mean sprint speed (*ρ* = −0.813, *p* = 0.008), while flexion showed significance only within the flexion-extension balance model (*β* = −0.68, *p* = 0.049). No significant correlations with mean sprint speed were observed for shoulder abduction or adduction PT/BW, and neither bilateral asymmetry nor agonist/antagonist balance showed significant main effects or interactions with PT/BW in the regression models.

**Conclusion:**

These findings suggest that, in WR athletes with cervical spinal cord injury, sprint performance may depend more on the available shoulder force-generating capacity than on the degree of bilateral symmetry or agonist/antagonist balance measured under controlled isokinetic conditions. From an applied perspective, strength and conditioning programs should prioritize functional torque production while interpreting asymmetries within each athlete's impairment-specific neuromuscular profile rather than treating symmetry restoration as a universal training target.

## 1 Introduction

Wheelchair Rugby (WR) is a Paralympic sport practiced by athletes with impairments affecting the upper and lower limbs, including reduced voluntary muscle strength, limb length, passive range of motion, or coordination (1–4). Athletes are assigned functional classes ranging from 0.5 to 3.5 points, reflecting differences in their functional abilities and influencing their tactical roles and wheelchair configurations during competition (5). WR is characterized by repeated short-duration, high-intensity efforts, with athletes typically performing 20–40 brief sprints of 2–4 s per game (4, 6), interspersed with collisions and rapid directional changes. Consequently, the ability to generate force rapidly is essential for gaining positional advantage and contributing to successful performance (7). The shoulders joint complex plays a central role in wheelchair propulsion, although propulsion strategies vary according to athletes’ functional profiles. High-class players predominantly use pushing movements supported by trunk and triceps function, whereas low- and middle-class players tend to rely more on pulling patterns involving the biceps (8). These differences highlight how athletes adapt their movement strategies to their individual neuromuscular impairments and functional capabilities to maintain propulsion efficiency and meet the demands of competition (5, 7–9).

Such individual adaptations raise important questions regarding the role of bilateral muscle symmetry in wheelchair propulsion and sport performance. Although greater bilateral symmetry has been associated with improved propulsion characteristics, including speed, acceleration, and mechanical efficiency (10), inter-limb asymmetry is increasingly recognized as a task-specific phenomenon influenced by the mechanical and technical demands of each sport and the athlete's functional profile. Evidence from wheelchair sports indicates that asymmetry varies across disciplines, reflecting differences in impairment characteristics, unilateral technical actions, and sport-specific movement strategies (11, 12). Furthermore, asymmetry is not necessarily a stable characteristic, as its magnitude and direction may vary over time and across movement tasks as athletes adjust their motor strategies to changing mechanical and tactical demands. Therefore, bilateral differences should not automatically be interpreted as performance deficits, particularly in athletes whose movement patterns are shaped by impairment-related constraints and functional adaptations. Furthermore, asymmetry is not necessarily a stable characteristic, as its magnitude and direction may vary over time and across movement tasks as athletes adjust their motor strategies to changing mechanical and tactical demands. Therefore, bilateral differences should not automatically be interpreted as performance deficits, particularly in athletes whose movement patterns are shaped by impairment-related constraints and functional adaptations.

The interpretation of inter-limb asymmetry is further complicated by limitations in commonly used quantification methods. Fixed thresholds, such as 10%–15%, have been widely adopted in able-bodied sports (13), but their universal application may overlook individual and task-specific characteristics. For example, longitudinal research in elite youth tennis players reported asymmetry magnitudes ranging from 2.0% to 15.9% across unilateral strength, power, speed, and agility assessments, while the direction of asymmetry was more consistent in the dominant upper limb than in the lower limbs (14). These findings suggest that asymmetry magnitude alone may not adequately represent its functional significance. Similarly, change-of-direction asymmetry, defined as the difference in performance between directional movement tasks, has been shown to decrease with chronological age, while the direction of asymmetry demonstrated low consistency across repeated assessments in youth elite tennis players (15). Together, these findings highlight that asymmetry is influenced by the assessment task, its magnitude and direction, and its consistency over time. These considerations are particularly relevant in Paralympic sports, where impairment-related neuromuscular variability, compensatory strategies, and functional constraints further influence movement patterns (9). Accordingly, a more comprehensive approach to investigating asymmetry in WR should consider not only the magnitude of bilateral differences but also their potential relationship with muscle function and sport-specific performance.

Although the relationship between inter-limb asymmetry and athletic performance has been extensively investigated, evidence remains inconsistent and dependent on the population, task, and asymmetry metric used (16–18). Although the relationship between inter-limb asymmetry and athletic performance has been extensively investigated, evidence remains inconsistent and dependent on the population, task, and asymmetry metric used (16–18). Compensatory strategies may allow athletes to maintain performance despite substantial bilateral differences, suggesting that asymmetry is not inherently detrimental (17, 19). This may be particularly relevant in wheelchair court sports, where asymmetries can reflect impairment-related constraints, technical demands, or functional adaptations rather than maladaptive deficits (20). In addition to bilateral asymmetry, the balance between agonist and antagonist muscle groups may provide complementary information about shoulder function. Agonist/antagonist ratios describe the relative torque-generating capacity of opposing muscle groups and have been used to characterize muscle function in performance and rehabilitation contexts (21–23). In WR, the shoulder musculature is repeatedly loaded during propulsion, stabilization, and sport-specific actions (24). Thus, differences in the relative capacity of opposing muscle groups may be relevant to understanding shoulder function and force production during these activities. However, the extent to which bilateral asymmetry and agonist/antagonist muscle balance are associated with sprint performance or modify the relationship between shoulder muscle strength and performance, remains insufficiently explored in WR.

Thus, the aim of this study were to: (1) investigate the presence and magnitude of bilateral asymmetry and imbalances in the agonist/antagonist ratio of shoulder muscles; (2) examine the association between peak torque in different shoulder movements and performance in the 20-m sprint test; and (3) analyze whether bilateral asymmetry and muscle balance influence 20-m sprint performance and interact with peak torque variables in predicting this outcome.

## 2 Methods

### 2.1 Study design and sample characterization

This exploratory, cross-sectional preliminary study included a convenience sample of WR athletes comprising all players who were available and eligible during the data-collection period. No *a priori* sample-size calculation was performed; therefore, the analyses should be interpreted as exploratory. Participants were required to be at least 18 years old, actively engaged in WR, and officially classified for competition. Exclusion criteria were shoulder muscle or joint injuries that prevented testing; restricted shoulder range of motion that precluded completion of the required movement arc; acute clinical conditions, including infection, autonomic dysreflexia, or extreme fatigue; surgery during the preceding six months; and use, at the time of assessment, of substances or medications that could alter strength, coordination, or performance, including anabolic steroids, high-dose caffeine supplements, methylphenidate-containing medication, benzodiazepines, or alcohol. All participants volunteered and provided written informed consent before prior to participation.

According to the training- and performance-caliber framework proposed by McKay et al. (22), nine highly trained national-level WR athletes with cervical spinal cord injury at neurological levels C5–C7 participated in this study. Seven athletes had complete injuries and two had incomplete injuries, resulting in heterogeneous preservation of upper-limb muscle function. Most participants retained functional triceps activation, whereas two lacked effective triceps function, a profile commonly observed among lower-point WR players. Functional classification ranged from 0.5 to 3.5 points, representing a broad spectrum of neuromuscular impairment and compensatory movement strategies. All participants trained regularly, completing approximately three two-hour on-court sessions per week. This functional diversity provides important context for interpreting the strength, asymmetry, and sprint-performance findings. Detailed participant characteristics are presented in Table 1. The study protocol was approved by the Research Ethics Committee of the State University of Campinas (CAAE: 70205323.9.0000.5404).

**Table 1:** Sample characterization.

Athlete*	Age (y)	Weight (Kg)	Height (cm)	Class
1	50	65.57	170	0.5
2	38	77.59	190	0.5
3	31	65.07	178	2.0
4	30	48.72	155	2.0
5	39	67.90	171	2.0
6	42	71.78	170	2.0
7	36	75.35	170	2.5
8	54	79.86	185	3.0
9	34	63.71	104	3.5
Mean ± sd	39.33 ± 7.70	68.39 ± 8.84	165.89 ± 23.85	

Y, years, Kg, kilogram, cm, centimeters.

*All participants had high-level spinal cord injury.

### 2.2 Experimental procedures

The tests were conducted on two days in the same week, with a one-day interval. In the first session, the athletes had their body length measured while lying in the supine position using a horizontal anthropometric ruler (Model 205HR, Health O Meter Professional, McCook, IL, USA) following the guidelines described in (25), and their body weight was measured with a wheelchair-accessible digital platform scale (LD1050, Líder Balanças, Brazil). Body length and weight values ​​were measured for entry into the isokinetic dynamometer software. The software uses this data to calculate relative force, apply gravity correction, and compare results against normative standards (26). Then, the participants were positioned in isokinetic dynamometer (Biodex System 4 Pro Multi-Joint System, Biodex Medical Systems, Inc., Shirley, NY, USA) to objectively and directly measure the performance and muscle function of the shoulder joint, including identification of potential imbalances and muscle deficits. In the second evaluation session, the athletes performed the 20-meter sprint test to measure speed in meters per second (m/s) and the total time to complete the test. A photocell system (Speed Test 6.0 Telemetric, CEFISE Biotecnologia Esportiva, Nova Odessa, Brazil) was used for this test. Athletes were instructed to maintain healthy eating habits, proper hydration, and to get 6 to 8 h of sleep each night throughout the evaluation week. During this period, participants refrained from engaging in physical or on-court training sessions. These standardized procedures were adopted to minimize confounding variables related to metabolic and neuromuscular fatigue, ensuring optimal data reliability, maximizing intra-subject reproducibility, and guaranteeing that the physical performance reflected the participants’ true maximal neuromuscular capacity (27–29).

#### 2.2.1 Isokinetic strength test

Shoulder flexion, extension, abduction, and adduction were evaluated at an angular velocity of 180°/s. This velocity represents an intermediate condition between maximal strength and muscular power, allowing a functional assessment aligned with the fast and repetitive demands of WR (13, 30). Intermediate velocities such as 180°/s are widely used in sports assessments because they balance torque production and movement speed, and have demonstrated sensitivity for detecting muscular asymmetries and imbalances (31, 32).

Participants were positioned in the Biodex System chair following manufacturer guidelines. The trunk was stabilized using three non-elastic straps: one across the chest, one across the pelvis, and one across the thighs, preventing compensatory trunk movements. The tested arm was secured to the dynamometer lever arm using a padded glove and Velcro straps to ensure consistent hand placement and prevent slippage.

For alignment, the dynamometer axis was carefully positioned according to anatomical landmarks specific to each movement: Flexion/extension: aligned with the humeral head, approximating the glenohumeral joint center; and abduction/adduction: aligned with the acromion process in the frontal plane. These landmarks were used to standardize joint alignment across participants. The seat height, backrest angle, and lever-arm length were adjusted individually and recorded to ensure consistent positioning between movements and across participants.

All analyses were restricted to the isokinetic range, defined as the portion of the movement in which the dynamometer maintained the preset velocity. The range of motion (ROM) for each movement was established according to Biodex System 4 manufacturer guidelines and adjusted individually to accommodate functional limitations of each athlete. For shoulder flexion and extension, the dynamometer axis was aligned with the humeral head, and ROM was set from approximately 0° (arm alongside the trunk) to 90° of flexion, respecting each athlete's available active range. For abduction and adduction, the axis was aligned with the acromion, and ROM was configured from 0° (arm in neutral position) to approximately 80–90° of abduction, again adjusted when necessary to avoid pain, compensatory trunk movement, or restrictions caused by spasticity or contractures. Seat height, backrest angle, and lever-arm length were standardized according to Biodex recommendations and recorded for each athlete. Trunk stabilization was ensured using chest, pelvic, and thigh straps to minimize compensatory movements

All assessments were conducted by the same researcher, who was formally trained in isokinetic testing procedures and familiarized with the Biodex system to ensure consistency across sessions. Before each test, participants received standardized instructions regarding the beginning and end of the movement, as well as the required effort level. During the assessment, real-time visual feedback from the dynamometer monitor was provided to help participants maintain the prescribed movement pattern. Verbal encouragement was offered throughout all repetitions to promote maximal and consistent effort, following established recommendations for isokinetic performance testing.

For flexion/extension, athletes performed five warm-up/familiarization repetitions followed by ten test repetitions (33). For shoulder abduction/adduction, three warm-up repetitions and five test repetitions were performed (34). Warm-up and test sets were separated by 60 s of rest, with 3 min between different movements. Verbal encouragement and real-time visual feedback were provided throughout testing.

Peak torque normalized by body weight (PT/BW) and the agonist/antagonist ratio were used for analysis. PT/BW allows comparison between individuals of different body sizes and is widely used as a performance indicator and return-to-sport criterion (26, 35). The agonist/antagonist ratio reflects the balance between opposing muscle groups and is commonly used in performance and injury-risk assessments (21, 22).

#### 2.2.2 Performance test

Sprint performance was assessed using a 20-m sprint test. For this test, a system with four photocells was used: two positioned side-by-side 50 cm apart, and two others positioned 20 meters away from the first two photocells, also side-by-side 50 cm apart. This configuration provided two photocell portals indicating the start and end of the test (Figure 1). The athletes were positioned in a line at the initial portal. Upon crossing this portal, the test was automatically initiated by the photocell system, and the athlete then propelled their wheelchair 20 meters as fast as possible, crossing the final portal and completing the test. The command to start the test was given by the researcher, and two attempts were performed with 5 min of rest between them. For analysis purposes, speed values were extracted, and the best result among the attempts was used.

**Figure 1:**
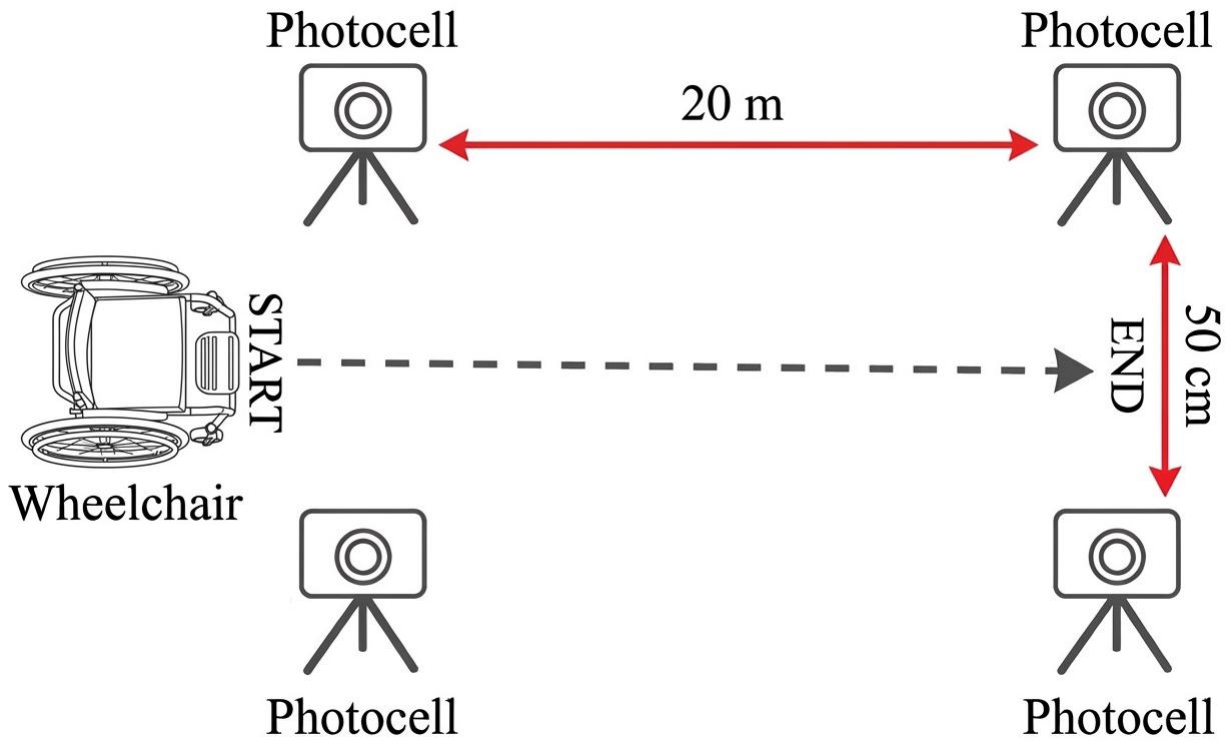
20 m sprint test. Configuration of the 20 m sprint test, with photocells positioned at the start and end of the course to record the test time and speed in meters per second.

### 2.3 Statistical analysis

Descriptive statistics, including mean and standard deviation, were utilized to characterize the sample. The Shapiro–Wilk test was applied to assess normality of the data distribution; however, given the small sample size, normality results were interpreted cautiously and did not determine the choice of statistical procedures. Instead, the analytical approach was selected according to the objectives of each analysis and the characteristics of the data. Spearman's rank correlation was selected because it does not require normally distributed variables and is suitable for assessing monotonic associations. Multicollinearity was addressed separately in the regression models by centering the predictor variables before creating interaction terms. This provided a conservative approach for assessing associations in this small sample, while recognizing that the assumptions relevant to the parametric analyses were considered separately.

Bilateral asymmetry was quantified as a continuous variable representing the magnitude of the percentage difference in peak torque between the right and left limbs, calculated using the absolute asymmetry formula (16):

$$Asymmetry\;\left(\%\right)=\frac{stronger\;side\;-\;weaker\;side}{stronger\;side}\;x\;100$$

No predefined threshold was applied to classify athletes as asymmetrical or symmetrical, as there is currently no universally accepted cutoff for interpreting inter-limb asymmetry across athletic populations. Therefore, all asymmetry values were retained as continuous measures in the statistical analyses. To investigate the presence and magnitude of bilateral asymmetry and agonist/antagonist imbalance (Objective 1), one-way ANOVA was performed to compare asymmetry indices between shoulder movements, followed by Tukey's *post-hoc* test when appropriate. Agonist/antagonist ratios were described and compared with accepted reference ranges to identify deviations from normative values: Flexion/Extension∼0.66–0.75, and Abduction/Adduction∼0.8–1.2 (13, 23).

To examine the association between PT/BW and sprint performance (Objective 2), non-parametric Spearman correlation coefficients were calculated. This approach is recommended for small samples and avoids assumptions of normality and multicollinearity. Correlations were computed between mean sprint speed and PT/BW values, bilateral asymmetry indices, and agonist/antagonist ratios. To analyze whether bilateral asymmetry and muscle balance (agonist/antagonist ratio) influence 20 m sprint performance and whether they interact with PT/BW variables in predicting mean sprint speed, (Objective 3), univariate linear regression models with interaction terms were fitted. Each model included one centered PT/BW variable, one centered asymmetry or agonist/antagonist variable, and their interaction. Centering was applied to reduce non-essential multicollinearity. The significance of interaction terms was evaluated using model coefficients and likelihood ratio tests comparing models with and without interaction. This analytical strategy is considered appropriate for ultra-small samples and avoids overfitting associated with multivariable regression. For the analysis examining associations with sprint performance (Objectives 2 and 3), the mean of the right- and left-arm values was calculated for PT/BW and agonist/antagonist ratios, because limb dominance was not an investigated factor. Thus, one value per movement was used for each of these variables in the correlation and regression analyses.

Effect sizes (Spearman's rho and standardized regression coefficients) were interpreted according to conventional benchmarks: small (≈0.10), moderate (≈0.30), and large (≥0.50), following Cohen's recommendations (36). A *post hoc* power analysis was conducted using G\*Power (version 3.1.9.7) to contextualize the inferential results obtained from the small sample. Power was calculated based on the observed effect sizes of the significant tests (correlation and regression), using *α* = 0.05 and the total sample size (*N* = 9). For the correlation analysis, the observed Spearman coefficient was entered as the effect size (approximated to Pearson's r for power estimation). For the regression model, the effect size was computed as $f^{2}=\frac{R^{2}}{1-R^{2}}$, following Cohen's conventions. Power values were interpreted according to standard benchmarks, where values ≥ 0.80 indicate adequate statistical power for detecting the observed effects (37). Statistical significance was set at *p* ≤ 0.05. All analyses were conducted using RStudio (version 4.4.1).

## 3 Results

Table 2 summarizes the descriptive statistics (mean, standard deviation, minimum, and maximum values) for all study variables.

**Table 2:** Descriptive statistics of isokinetic variables by movement and limb (right and left arms), and sprint performance (*n* = 9).

Isokinetic shoulder variables	Right Arm		Left Arm	
	Mean ± SD	Min – Max	Mean ± SD	Min– Max
PT/BW Flexion (Nm/kg)	102.9 ± 22.6	73.1–138.6	98.2 ± 27.4	54.2–141.5
PT/BW Extension (Nm/kg)	63.8 ± 25.3	30.5–113.6	62.0 ± 18.7	38.3–98.1
Flexion/Extension Ratio	171.7 ± 52.4	88.0–245.4	165.1 ± 41.3	109.6–238.9
PT/BW Abduction (Nm/kg)	100.2 ± 52.1	32.7–212.0	98.0 ± 31.8	33.6–171.7
PT/BW Adduction (Nm/kg)	47.0 ± 20.0	11.9–94.2	45.6 ± 16.4	15.8–67.2
Abduction/Adduction Ratio	210.7 ± 45.1	163.0–275.0	214.0 ± 46.2	182.6–307.5
Sprint performance	Mean ± SD		Min – Max	
20-m Sprint Time (s)	6.72 ± 1.10		5.5–9	
20-m mean sprint speed (m/s)	3.05 ± 0.42		2.23–3.63	

Bilateral asymmetry indices based on PT/BW values varied among the different shoulder movements. The highest asymmetry magnitude was observed for the adduction movement (mean = 25.78%; sd = 13.54), followed by extension (mean = 17.01%; sd = 12.75) and flexion (mean = 14.69%; sd = 7.24). Shoulder abduction showed the lowest asymmetry magnitude (mean = 9.03%; sd = 5.87). ANOVA indicated a significant difference in asymmetry levels between shoulder movements (F_(3,32)_ = 4.03, *p* = 0.015). Tukey's *post-hoc* test revealed that asymmetry in adduction was significantly greater than in shoulder abduction (difference = 16.75%, *p* = 0.009). No other comparison between movements showed a statistically significant difference (Figure 2).

**Figure 2:**
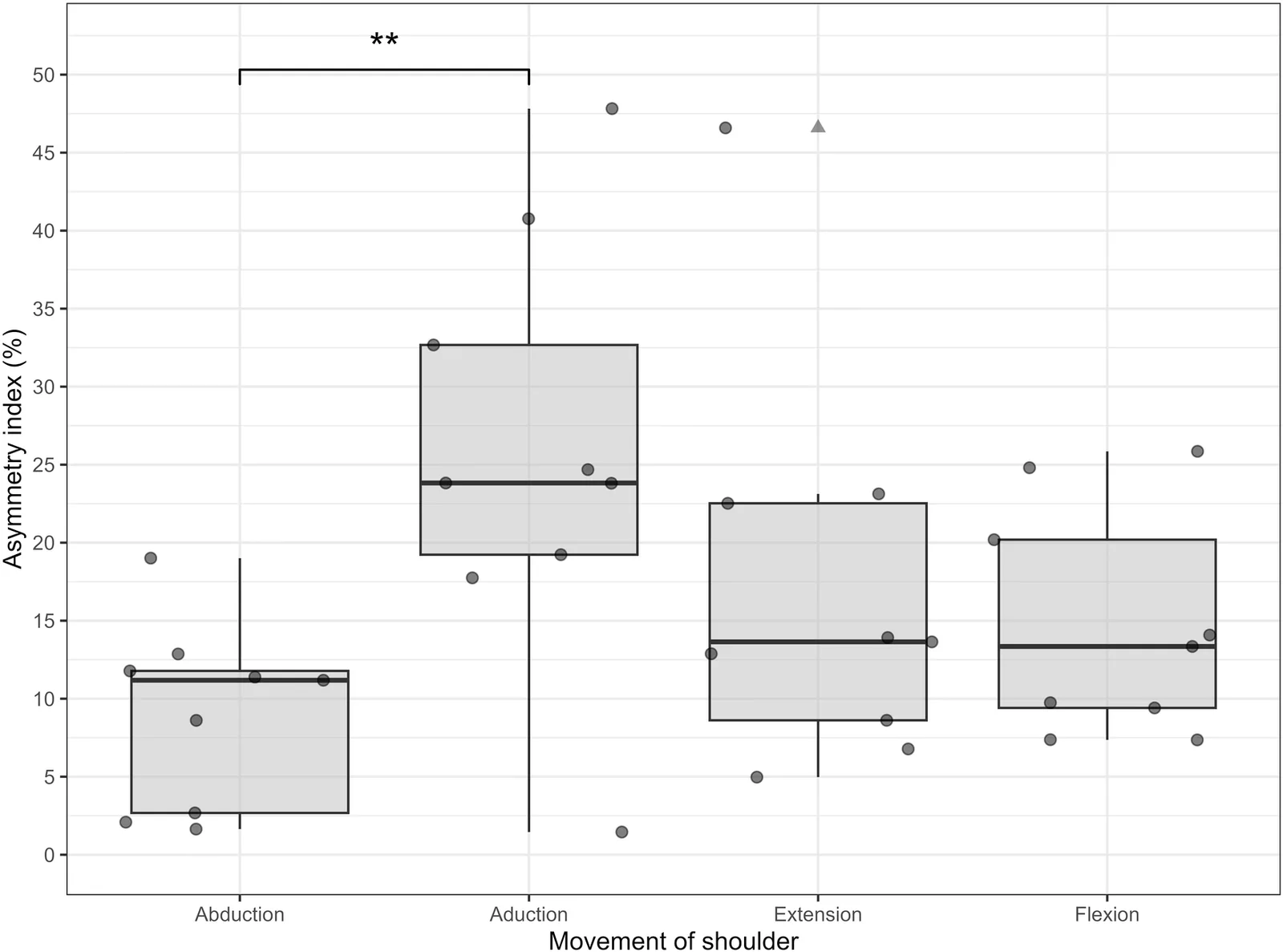
Bilateral shoulder asymmetry index by movement. Black points correspond to individual participants, and the red triangle indicates an outlier. A significant difference was observed between abduction and adduction (*p* < 0.01).

The agonist/antagonist ratio showed values higher than the normal ranges. For the flexion/extension movement, the observed average was 1.69 ± 0.43, representing a positive deviation of +0.98 from the central value of the reference range (0.705) (13, 23). This result indicates a marked predominance of flexor muscles compared to extensors. In the shoulder abduction/adduction movement, the average was even higher (2.26 ± 0.32), corresponding to a deviation of +1.26 from the reference value (1.00) (13, 23). This finding demonstrates a muscle imbalance, with predominance of abductors over adductors see Figure 3.

**Figure 3:**
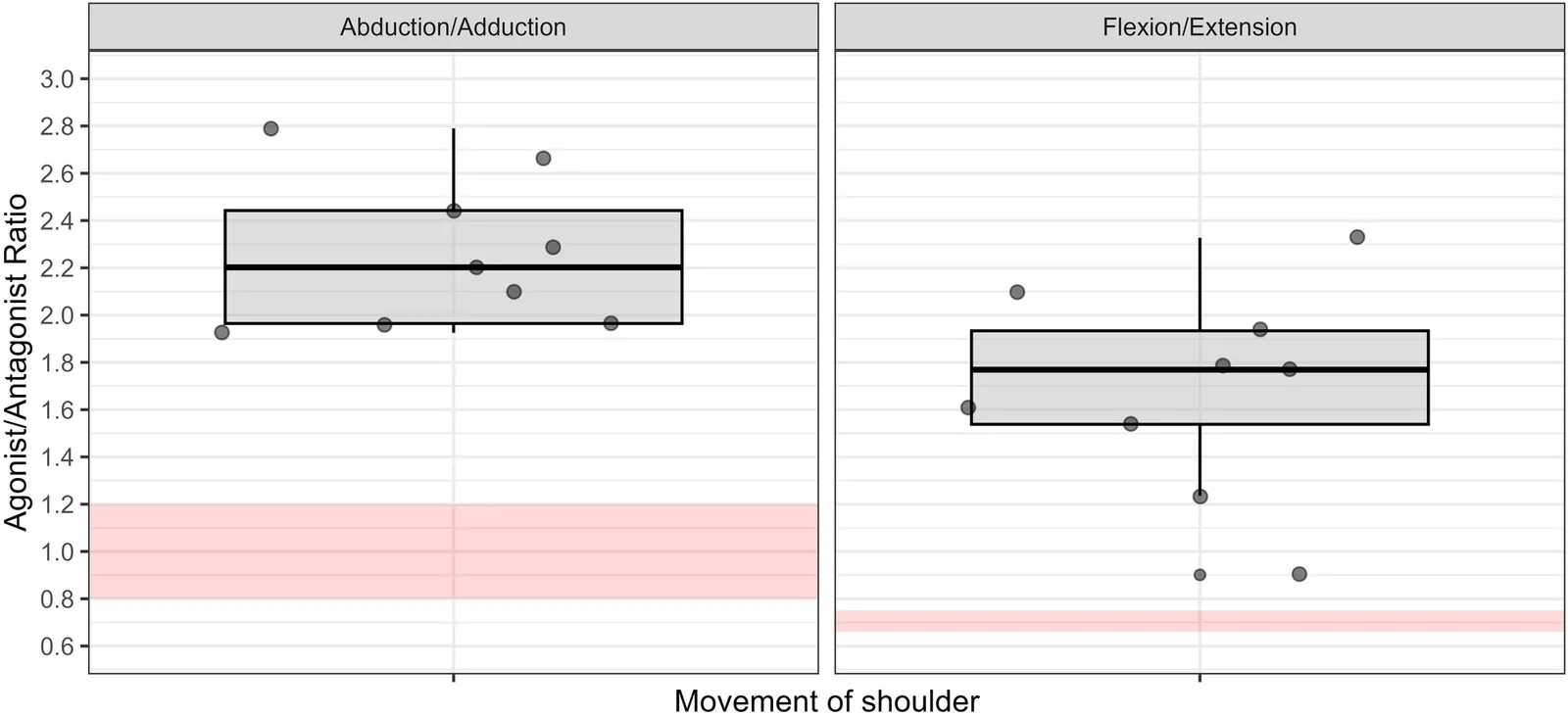
Agonist/Antagonist Ratio by Movement. Distribution of the agonist/antagonist ratio for shoulder abduction/adduction and flexion/extension movements. Each boxplot represents the variability of the athletes in each movement, including median, quartiles, and individual values ​​(points). The area shaded in red indicates the clinical range of normality described by Dvir (2004), used as a reference to identify muscle imbalances. Values ​​above this range represent a predominance of the agonist group in relation to the antagonist.

Spearman correlation analysis showed that shoulder extension PT/BW was significantly and positively associated with mean sprint speed (*ρ* = 0.812, *p* = 0.008). No other isokinetic variable demonstrated a significant correlation with sprint performance. In the univariate regression models, peak torque in shoulder flexion was the only variable significantly associated with mean sprint speed (*p* = 0.049) when examined alongside the flexion–extension agonist/antagonist ratio. Neither bilateral asymmetry nor muscle balance showed significant main effects or interaction effects in any model. Additionally, *post hoc* power analysis demonstrated adequate statistical power for the significant associations, with powe*r* = 0.88 for the correlation between extension PT/BW and mean sprint speed, and powe*r* = 0.85 for the regression coefficient of shoulder flexion PT/BW. Associations between isokinetic variables and sprint performance, together with the muscle asymmetry comparisons, are presented in Table 3.

**Table 3:** Inferential analyses of the associations between isokinetic variables and sprint performance, and comparisons of muscle asymmetry.

Variable	Analysis	Test statistic	*p* value
Association with 20-m sprint time			
PT/BW – Flexion	Spearman correlation	*ρ* = 0.320	0.390
PT/BW – Extension	Spearman correlation	ρ = 0.812	0.008
PT/BW – Abduction	Spearman correlation	ρ = 0.180	0.620
PT/BW – Adduction	Spearman correlation	ρ = 0.220	0.540
PT/BW – Flexion	Linear regression	*β* = −0.68	0.049
PT/BW × Asymmetry	Likelihood ratio test	-	> 0.05
PT/BW × Agonist/Antagonist ratio	Likelihood ratio test	-	> 0.05
Shoulder muscle asymmetry comparisons			
Overall comparison	One-way ANOVA	F_(3,32)_ = 4.03	0.015
Adduction vs. Abduction	Tukey *post hoc*	*Δ* = 16.75%	0.009

* statistically significant values (*p* ≤ 0.05).

## 4 Discussion

The present study aimed to: 1) investigate the presence and magnitude of bilateral asymmetry and imbalances in the agonist/antagonist ratio of shoulder muscles in WR athletes; 2) examine the association between the muscle peak torque of different shoulder movements and performance in the 20 m sprint test; and 3) analyze whether bilateral asymmetry and muscle balance influence performance in the 20 m sprint and whether they interact with muscle peak torque variables in predicting this performance. The results showed that the magnitude of bilateral peak torque differences varied across athletes and shoulder movements, particularly in shoulder adduction and extension movements, and that all exhibited marked imbalances in agonist/antagonist ratios, with a predominance of flexors and abductors. Despite this asymmetrical neuromuscular profile, the Spearman correlation analysis indicated that the averaged shoulder extension PT/BW (mean of both limbs) was significantly associated with mean sprint speed. However, in the univariate regression models, shoulder flexion PT/BW was the only variable significantly associated with mean sprint speed when examined alongside the flexion–extension agonist/antagonist ratio. Neither bilateral asymmetry nor muscle balance showed significant main effects or interaction effects in this sample.

Inter-limb asymmetry is a common finding across athletic populations and has been described as a sport-specific characteristic influenced by the demands of each sport (16, 38–41). However, previous evidence indicates that the magnitude of asymmetry varies considerably depending on the sport and assessment method, and no universal threshold exists to classify asymmetry as low or high (16, 38–41). Although fixed asymmetry thresholds are frequently criticized, some studies have used population-specific thresholds based on group means or medians, attempting to contextualize asymmetry within the functional characteristics of a given athletic cohort (42, 43). In the present study, the pronounced asymmetry and marked muscle imbalances is compatible with the functional profile of tetraplegic athletes and aligns with previous descriptions of neuromuscular adaptations following cervical spinal cord injury. Disruption of motor and sensory pathways may result in reduced muscle activation, selective muscle loss, altered motor-unit recruitment, and uneven preservation of agonist and antagonist muscles, promoting compensatory movement patterns and asymmetrical loading and functional reorganization of movement (44–47). These factors result in highly individualized motor patterns, often marked by biomechanical compensations to maintain functionality (48).

Such compensatory strategies are particularly relevant in WR, where the upper limbs perform functions normally shared with the lower limbs, including propulsion, braking, directional changes, postural stabilization, and sport-specific technical actions (4). In athletes with cervical spinal cord injury, weakness or absence of the triceps may increase the contribution of the shoulder musculature to both propulsion and stabilization during the push phase (46, 47). The latissimus dorsi and posterior deltoid, primary extensors of the shoulder, contribute to controlling the terminal portion of the push and to repositioning the arm during the recovery phase, which is essential for maintaining propulsion cadence at high speeds (47). In this context, the significant association between shoulder extension torque and sprint performance may reflect the importance of the extensors in controlling the terminal push and repositioning the arm during recovery, thereby supporting propulsion cadence at high speeds. Although shoulder flexion is also involved in forward propulsion, its contribution may be partly overshadowed by the stabilizing and control demands placed on the extensors during high-speed propulsion.

Despite the marked agonist/antagonist imbalances observed, particularly the predominance of flexors over extensors and abductors over adductors, these structural deviations did not translate into measurable effects on sprint performance. This absence of association was somewhat unexpected, given that more balanced flexion–extension profiles could theoretically enhance mechanical efficiency during propulsion. One possible explanation is that WR athletes with cervical SCI rely heavily on compensatory strategies that redistribute mechanical demands across available muscle groups (49), allowing them to maintain propulsion effectiveness even in the presence of substantial imbalances. Thus, although extension torque emerged as a key determinant of sprint performance, the underlying imbalance between flexors and extensors did not appear to constrain performance in this specific task. Consistent with this interpretation, flexion torque did not correlate significantly with sprint performance but remained relevant in the regression model, supporting its contribution to forward propulsion (50, 51). When the hand is fixed on the rim, shoulder flexor activation contributes to forward translation of the arm–trunk segment and indirectly facilitates the elbow extension required to complete the push phase (23). Nevertheless, shoulder flexion and abduction did not demonstrate independent associations with mean sprint speed, suggesting that their contribution may be overshadowed by the stabilizing and control demands placed on the extensors during high-speed propulsion.

Importantly, neither bilateral asymmetry nor muscle imbalance influenced sprint performance or modified the relationship between torque and mean sprint speed. Even though shoulder adduction exhibited significantly greater asymmetry compared to abduction, this structural imbalance did not translate into functional impairment. This finding supports the view that neuromuscular variability and compensatory activation strategies may allow athletes to maintain functional performance despite inter-limb asymmetries, emphasizing available functional capacity rather than symmetry alone (52). However, given the limited statistical power, this finding should be interpreted cautiously and does not rule out potential relevance of symmetry in other contexts or populations. Previous research has demonstrated that force application varies significantly from one push to another (cycle-to-cycle variability), indicating that the motor system is constantly readjusting to maintain functional performance, making constant symmetry a less realistic goal (24). Interestingly, previous studies have reported that high-point wheelchair court athletes, who are typically the fastest and most powerful, tend to exhibit greater asymmetries than low-point athletes. This pattern is thought to arise because having more functional muscle mass increases the potential for strength differences between upper limbs, especially in sports with substantial unilateral loading (7).

Although symmetry and muscle balance have traditionally been regarded as desirable characteristics for athletic performance and injury prevention (53, 54), current evidence indicates that their functional consequences are highly context-dependent. The relationship between asymmetry, performance, and injury varies according to the population, sport, task, and method used to quantify asymmetry (18, 55). This is particularly relevant in athletes with neurological impairments, in whom asymmetry may reflect the underlying impairment and long-term neuromuscular adaptations rather than solely maladaptive characteristics. Supporting this interpretation, Chapelle et al. (11) found only weak associations between morphological and functional asymmetries in elite youth tennis players, suggesting that structural asymmetry does not necessarily result in functional deficits. Systematic reviews have similarly reported inconsistent associations between inter-limb asymmetry and both performance and injury risk (18, 55), while D’Hondt et al. (56) demonstrated that different asymmetry measures may yield substantially different relationships with performance. These findings, together with evidence that fatigue-related changes in asymmetry are task-dependent (56), reinforce the dynamic and context-specific nature of asymmetry.

In the present study, despite the presence of considerable inter-limb asymmetry and agonist-antagonist imbalance, PT/BW remained a significant determinant of sprint performance. These findings suggest that force-generating capacity may be more relevant to short-distance wheelchair propulsion than the asymmetry measures evaluated in this study. The lack of association between muscle balance and mean sprint speed was somewhat surprising, particularly given the pronounced predominance of flexors over extensors and abductors over adductors. However, athletes with cervical spinal cord injury often develop long-term compensatory strategies that allow them to generate effective propulsion despite substantial deviations from normative balance ranges (49). These compensations may buffer the functional consequences of muscle imbalance during short, maximal sprints, where the ability to generate rapid force may outweigh the mechanical disadvantages associated with disproportionate agonist/antagonist profiles. From a practical perspective, strength and conditioning programs for athletes with tetraplegia should therefore prioritize functional force production and torque-generating capacity while considering each athlete's individual neuromuscular profile. Rather than viewing asymmetry solely as a deficit requiring correction, practitioners may consider it a window of opportunity to identify individual neuromuscular characteristics, guide individualized training interventions, and monitor changes over time (16, 38–42). In this context, the presence and magnitude of asymmetry should be interpreted alongside functional performance and the athlete's individual profile, distinguishing potentially maladaptive asymmetries from stable neuromuscular adaptations associated with cervical spinal cord injury and long-term sport participation (42).

This study has several limitations that must be considered when interpreting the findings. The small sample size substantially limits statistical power and increases the likelihood of both type I and type II errors; therefore, non-significant results cannot be interpreted as absence of effect, and significant associations should be interpreted cautiously. The use of a convenience sample from a single elite WR team also restricts generalizability to other teams, competitive levels, and impairment profiles, reinforcing the preliminary and hypothesis-generating nature of the study. In addition, the absence of athletes representing all functional classes prevented stratified analyses, limiting the ability to examine how different levels of neuromuscular impairment influence strength, asymmetry, and performance. Although isokinetic testing provides controlled and reliable torque measurements, it does not fully capture the mechanical and neuromuscular demands of high-speed wheelchair propulsion, which involves technical variability, compensatory strategies, and game-specific actions. The absence of significant associations between asymmetry and sprint performance should not be interpreted as evidence that asymmetry is unrelated to performance in athletes with tetraplegia. Rather, it indicates that the specific asymmetry metrics investigated were not associated with this particular performance task. Other asymmetry assessments or sport-specific functional tests may reveal different relationships. Finally, the lack of information on shoulder injury history precluded exploration of potential relationships between asymmetry, muscle imbalance, and injury risk.

## 5 Conclusion

In conclusion, this preliminary study suggests that short-distance sprint performance in WR athletes with cervical spinal cord injury may be more closely related to the capacity to generate functional shoulder torque than to the magnitude of bilateral asymmetry or agonist/antagonist imbalance measured during isolated isokinetic testing. From a practical perspective, these findings support strength and conditioning approaches that prioritize the development and maintenance of shoulder force production, particularly within the movement strategies available to each athlete, rather than assuming that greater symmetry necessarily translates into better sprint performance. Scientifically, the results reinforce the need to interpret asymmetry in Paralympic athletes as a task- and impairment-specific characteristic that may reflect adaptive neuromuscular organization rather than a universal performance deficit. Because of the small and functionally heterogeneous sample, these conclusions should be considered exploratory. Future studies with larger, classification-stratified and longitudinal designs should determine whether specific asymmetry patterns become relevant under different performance tasks, fatigue conditions, or injury-risk contexts.

## Data Availability

The raw data supporting the conclusions of this article will be made available by the authors, without undue reservation.
